# Augmenting the Efficacy of a Polyvinyl Alcohol Selective Layer Coated on Polyvinylidene Fluoride Support Membranes with Kaolinite Introduction for Improved Pervaporation Dehydration of Epichlorohydrin/Isopropanol/Water Ternary Systems

**DOI:** 10.3390/polym16060835

**Published:** 2024-03-18

**Authors:** Shivshankar Chaudhari, YeWon Jeong, HyeonTae Shin, SeWook Jo, MinYoung Shon, SeungEun Nam, YouIn Park

**Affiliations:** 1 Department of Industrial Chemistry, Pukyong National University, San 100, Yongdang-dong, Nam-gu, Busan 608-739, Republic of Korea; 2 Center for Membranes, Korea Research Institute of Chemical Technology, 141 Gajeong-ro, Yuseong-gu, Daejeon 305-600, Republic of Korea

**Keywords:** mixed matrix membrane, kaolinite, exfoliation, hydrophilicity, epichlorohydrin, dehydration

## Abstract

Composite membranes with a polyvinyl alcohol (PVA) selective layer composed of well-dispersed hydrophilic kaolinite particles coated on a polyvinylidene fluoride (PVDF) support were developed. They were applied to the pervaporation dehydration of the industrially important epichlorohydrin (ECH)/isopropanol (IPA)/water ternary mixture. In comparison with raw kaolinite (RK), hydrophilic kaolinite (HK) enhanced the mechanical properties, hydrophilicity, and thermal stability of the PVA selective layer, as confirmed by universal testing, the contact angle, and TGA analyses, respectively. The pervaporation results revealed that the addition of HK particles significantly enhanced the separation factor (3-fold). Only a marginal reduction in flux was observed with ECH/IPA/water, 50/30/20 (*w*/*w* %) at 40 °C. An HK particle concentration of 4 wt.% with respect to PVA delivered the highest flux performance of 0.86 kg/m^2^h and achieved a separation factor of 116. The PVA–kaolinite composite membrane exhibited pronounced resistance to the ECH-containing feed, demonstrating a sustained flux and separation factor throughout an extended pervaporation stability test lasting 250 h.

## 1. Introduction

Epichlorohydrin (ECH) is a hazardous substance that contains chlorinated epoxy moiety [1,2], and isopropanol (IPA) is commonly used as a solvent in the epoxy resin manufacturing process. The water produced during synthesis forms an azeotropic ternary mixture with ECH and IPA [3,4]. Therefore, recycling (mainly dehydrating) this hazardous mixture is crucial for increasing product competitiveness in an environmentally friendly manner. However, the distillation process uses a lot of energy to separate zeotropic mixtures. The pervaporation process, on the other hand, due to its energy-efficient and ecologically friendly nature has sparked a lot of interest in treating a number of azeotropic mixtures [5,6]. Apart from the utilization of the third component in the pervaporation process, the more desirable component seeps preferentially from the liquid mixtures through a membrane [7], and thus, separation can be achieved.

Polyvinyl alcohol (PVA) is a biocompatible membrane-forming material with abundant hydroxyl functionalities in its linear aliphatic chains that can be easily crosslinked. Therefore, to date, PVA is the most preferable polymer for making membranes for pervaporation dehydration purposes [8,9,10]. However, owing to the inherent trade-off between flux and selectivity in PVA-based membranes, it is clear that there is still room for improvement in the performance of PVA membranes. Adopting nanomaterials as a filler in the PVA matrix, e.g., metal–organic frameworks [8,11], graphene oxide [12], MXene [10], carbon nitride [13], etc., has been investigated as a viable approach to simultaneously improve the total flux and separation factor. The choice of nanomaterial for integration into the PVA membrane is a critical consideration, as non-selective defects are likely to form in the membrane matrix due to the lack of interaction between the PVA matrix and filler [8,14].

Kaolinite is a 1:1 type layered silicate mineral known for its high surface area and interlayer spaces that offer the potential for improved membrane performance. One side of the kaolinite mineral is covered with the hydroxyl group of Al_2_(OH)_4_ octahedral sheets, and the other side is an oxygen atom of SiO_4_ tetrahedral sheets [15]. Due to the high strength of hydrogen bonding in the layer, exfoliation of kaolinite is necessary to obtain desirable properties for membrane application. The use of raw kaolinite generally results in a lower surface area and larger size, thereby increasing the tendency to aggregate in the polymer matrix. In this context, the exfoliation process, aimed at decomposing larger aggregates into smaller sizes, represents a viable approach to enhance the applicability of kaolinite in membrane applications. In this work, dimethyl sulfoxide is a polar molecule used as an intercalating/delaminating agent [16]. Through this approach, the hydrophilic properties of kaolinite can be improved by changing the Al/Si (aluminum to silicon) ratio in the layer. Furthermore, as a result of exfoliation, a certain degree of amorphicity is induced, imparting significant porosity to the kaolinite particles. Therefore, it can significantly enhance their dispersion in the PVA matrix by forming hydrogen bonds and providing smooth water transportation through the interlayer spacing. Moreover, the incorporation of exfoliated kaolinite into the PVA matrix is anticipated to enhance the mechanical and thermal properties, facilitating selective membrane transport and thereby contributing to enhanced stability.

Therefore, in this study, we focused on the development of a novel composite pervaporation membrane consisting of a PVA selective layer embedded with exfoliated kaolinite coated on a PVDF support. The utilization of such membranes in conjunction with the pervaporation technique offers an economically feasible and environmentally sustainable approach for the separation of water, isopropanol (IPA), and epichlorohydrin (ECH) mixtures. Based on the authors’ comprehensive literature review, no existing reports have been found detailing the application of exfoliated kaolinite within PVA membranes for the pervaporation dehydration of ECH–IPA–water mixtures. This research further discusses the preparation, characterization, and performance evaluation of the DMSO-exfoliated kaolinite-embedded PVA membrane for ECH/IPA/water pervaporation dehydration. Using systematic analysis, we aimed to investigate the impact of kaolinite content and the membrane structure on separation efficiency and flux. The findings of this study hold the potential to contribute to the development of advanced pervaporation membranes for the efficient and sustainable separation of challenging ECH/IPA/water mixtures, thus addressing critical industrial needs.

## 2. Experimental Methods

### 2.1. Materials

PVA (97,000 kg/mol, degree of hydrolysis 99%) and glutaraldehyde (25 wt.%) were procured from Alfa Aesar (Ward Hill, MA, USA). Kaolinite (quality level 100) and lithium chloride (99.99%) were purchased from Sigma-Aldrich (St. Louis, MO, USA). Dimethyl sulfoxide (DMSO), hydrochloric acid (HCl, 36.0%), ECH (99.0%), and IPA were purchased from Duksan Ltd. (Ansan, Republic of Korea). Details of the PVDF support preparation and characterization are provided in our previous study [17].

### 2.2. Preparation of Hydrophilic Kaolinite (HK)

LiCl was dissolved in DMSO by sonication to obtain a final concentration of 1 M. In the next step, 1 g of kaolinite particles were added to 40 mL of the LiCl (1 M) solution. Using an ultrasonicator (Branson Sonifier 450, Brookfield, WI, USA) at the 20% duty cycle, sonication was performed for 14 h at 80 °C. The HK colloidal solution was then centrifuged and washed successively with water and methanol. The obtained HK sediment was transferred to a vacuum oven and dried at 80 °C for 1 day.

### 2.3. Preparation of the PVA/HK-PVDF Membrane

In a clean glass bottle, 10 g of PVA was added to 90 g of water, and the mixture was subsequently transferred to an oven pre-set at 90 °C for one day. The mixture was then cooled to room temperature. In separate bottles, the dried HK powder was dispersed in water via sonication for 30 min. To obtain a coating solution with a final PVA content of 5 wt.%, 50 mL of the HK dispersion was added to 50 mL of the PVA solution. The HK concentration was varied from 2 to 15 wt.% with respect to PVA in the final solution to obtain different HK/PVA membranes. To these solutions, one drop of concentrated HCl and 0.25 g of glutaraldehyde was added, and the solution was stirred at 25 °C for 10 min. The different HK/PVA coating solutions were casted with an applicator (Bakers, Orlando, FL, USA) of 100 µm wet casting thickness on the PVDF support. The HK/PVA-PVDF membrane was dried in an oven at 30 °C (40% relative humidity (RH)) for one day. Subsequently, it was cured in the same oven for 1 h at 80 °C. For characterization purposes, free-standing films with 50 µm (confirmed on the thickness gauge (Mitutoyo, 2109S-10, Kawasaki, Japan)) of RK/PVA and HK/PVA were prepared by casting the above solution on a glass plate. Thereafter, a similar procedure was followed for curing.

### 2.4. Characterization Techniques for Kaolinite Particles and the PVA Membrane

Attenuated total reflection Fourier-transform infrared (ATR-FTIR) spectroscopy (Nicolet iS10, Waltham, MA, USA) was employed to investigate the chemical composition of the HK particles and kaolinite composite membranes. The samples were scanned in the range of 600–4000 cm^−1^ with a resolution wavenumber of 2 cm^−1^, and the FTIR spectra were recorded. The morphologies of the HK and RK particles and their PVA composites were observed using field-emission scanning electron microscopy (FESEM (VEGA-II LSU, TESCAN, Brno, Czech Republic). All the samples were gold-sputtered to make the surface conductive before testing. X-ray photoelectron spectroscopy (XPS) (Multi lab 2000, Shimadzu, Kyoto, Japan, and Kratos HP Thermo VG scientific, Oxford, UK, with a 200 W Al Kα monochromatic X-ray source) was used to observe the elemental composition of kaolinite and its core level chemistry. The crystalline nature of kaolinite powder was confirmed using an X-ray diffractometer (D/Max 2500, Rigaku, Tokyo, Japan). The surface area of the kaolinite particles was examined using a Brunauer–Emmett–Teller (Brunauer–Emmett–Teller) instrument (Quanta chrome autosorb IQ, Houston, TX, USA). To study their surface hydrophilicity, water contact angle measurements were performed on the kaolinite composite membranes. The sessile droplet method was used and, according to the shape of the water drops at the membrane surface (25 °C, 50% RH), the water contact angle was accounted on the contact angle analyzer (Phoenix 150, Waco, TX, USA). Thermogravimetric analysis (TGA) of different kaolinite–PVA composite membrane samples was performed using a TGA 7 instrument (Perkin Elmer, Waltham, MA, USA). The mechanical properties of the kaolinite–PVA composite membranes were determined using a universal testing machine (AGX-X, Shimadzu, Japan) equipped with Trapezium X software.

### 2.5. Degree of Swelling

The membrane samples were cut into 2 cm × 2 cm pieces, dried at 25 °C for 5 h in a vacuum oven, and weighed (*W_d_*) using an electronic balance. Then, they were immersed in 50 mL of water, the ECH and IPA, and feed solution, and put in an oven at 40 °C. After 24 h of swelling, the cells were removed, gently blotted with tissue paper to remove the residual mixture on the surfaces, and immediately weighed (*W_s_*). The swelling degree (*SD*) was calculated using Equation (1).

(1) $$SD\;\left(\%\right)=\frac{W_{s}-W_{d}}{W_{d}}\times 100,$$

where *W_s_* and *W_d_* are the masses of the swollen and dried membranes, respectively.

### 2.6. Pervaporation Experiment Procedure

PV separation of the ECH/IPA/water mixture was performed at various mixing ratios and feed temperatures (40–60 °C). The PV apparatus described in our earlier study [17] was used to conduct the PV tests in the laboratory. With an effective area of 0.001943 m^2^, the membrane was fitted to a stainless steel membrane cell with an effective area of 0.001943 m^2^. A peristaltic pump (YZ1515x, Longer Pump, Tucson, AZ, USA) was used to pump the feed mixture (70 mL/min) onto the membrane feed side. The pump was connected to a thermostatic bath to regulate the temperature of the feed solution. A vacuum pump (Super bee, USA; VOP-100, Vacuumer, Daegu, Republic of Korea) operating at a pressure of less than 1 Torr was used to maintain a vacuum on the permeate side of the membrane. The test was conducted under a vacuum for 5 h following saturation; the permeate sample was collected in traps that had been previously dipped in liquefied nitrogen. Equations (2) and (3) were used to calculate the flux (*J*) and separation factor (*α*) with the collected mixture. To improve accuracy, each membrane sample was tested three times, and the average result with a standard deviation of less than two was reported. The separation factor was determined using a gas chromatograph coupled with flame ionization detectors (DS7200, DS Science, Hwaseong, South Korea).

(2) $$J\;\;\frac{Q}{A\times t},$$

where *Q* (g), (*A* m^2^), and *t* (h) are the mass of the solution-permeating solution, effective membrane area, and time, respectively.

(3) $$\alpha =\frac{Pw/\left(Pe+Pi\right)}{Fw/Fe+Fi)},$$

where *Pw*, *Pe*, *Pi*, *Fw*, *Fe*, and *Fi* are the mass fractions of water, ECH, and IPA in the permeated and feed solutions, respectively.

## 3. Results and Discussion

### 3.1. Characterization of RK and HK Particles

**Figure 1:**
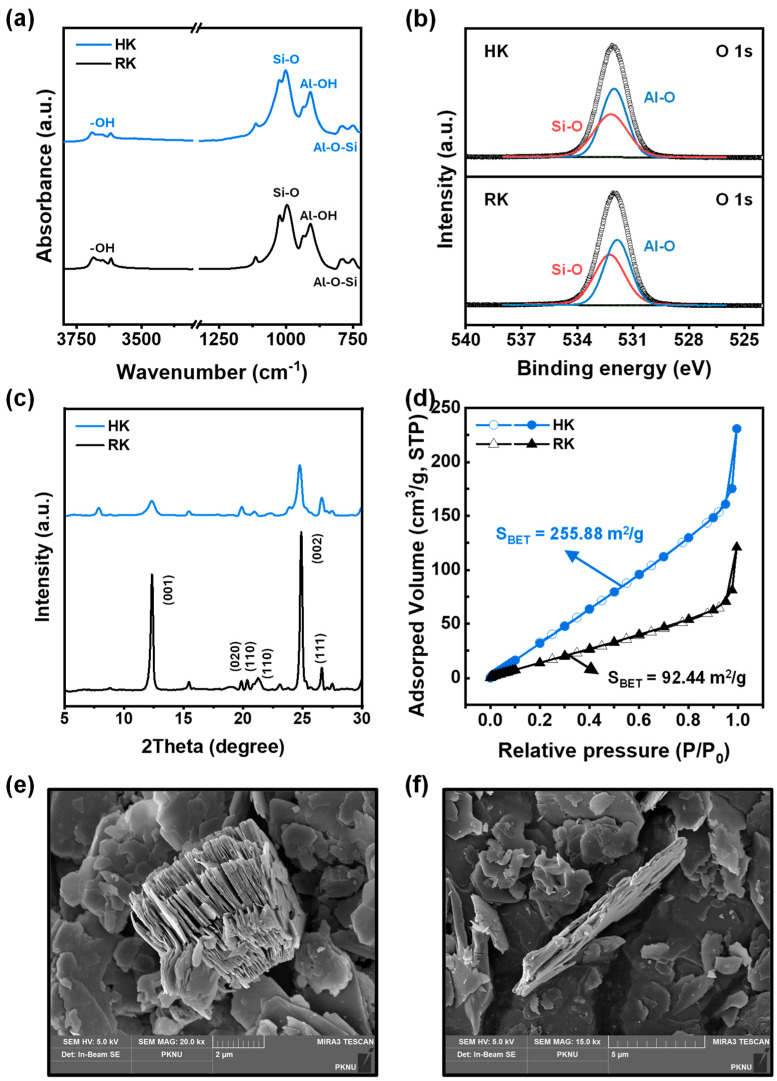
(**a**) FT-IR spectra, (**b**) O 1s XPS spectra, (**c**) XRD patterns, (**d**) BET analysis, and FESEM images of (**e**) RK and (**f**) HK particles.

The exfoliation of kaolinite particles with DMSO was examined using FTIR spectroscopy (Figure 1a). The FTIR spectra for both the RK and HK (DMSO-treated kaolinite) showed identical characteristic bands at 3685–3618 cm^−1^, which were attributed to the stretching of the inner surfaces of the OH groups [18,19]. The peaks at 1112, 1024, and 996 cm^−1^ corresponded to the stretching vibrations of Si–O–Si in the layer of kaolinite. Al–O–Si bending and Al–OH stretching vibrations were observed at 936, 909, and 791 cm^−1^ and 749 cm^−1^, respectively [16]. Additionally, owing to the weakening of the interlayer H-bonds and formation of new H-bonds between the kaolinite inner surface OH groups and the DMSO molecule, a new band appeared in the HK spectra at 3502 cm^−1^, suggesting successful exfoliation of the kaolinite particles by DMSO.

XPS analysis was conducted for RK and HK, and the atomic concentrations of the RK and HK particles are listed in Table 1. Upon DMSO exfoliation, the atomic concentrations of oxygen, silicon, and aluminum components remained largely unchanged, suggesting the preservation of the structural chemical composition of kaolinite. High-resolution O1s spectra of HK and RK are shown in Figure 1b. Peaks of Al–O and Si–O at binding energies of 531.85 and 532.25 eV, respectively, were observed in the RK spectra [20]. Notably, the Al–O peak shifted to 532.03 eV upon exfoliation, which represents the weaker hydrogen bonding interaction between the DMSO molecule and the OH groups of the Al_2_(OH)_4_ moieties in the octahedral structure. Furthermore, the Si/Al atomic ratio calculated from the XPS data of RK (1.18) was lower than that of HK (1.16) [21]. This indicates that the exfoliation of kaolinite particles changed the coordination number of Al and resulted in an increase in the hydrophilic Al content of the kaolinite particles.

XRD analyses of RK and HK were performed to identify changes due to exfoliation in the crystal plane of the triclinic crystal system (Figure 1c). For both RK and HK, the XRD curves exhibited diffraction peaks at 2θ = 12.34°, 19.85°, 20.35 and 21.24° and 24.89°, 26.60° corresponding to the (001), (020), (110), (002), and (111) crystallographic planes of kaolinite minerals, respectively [19]. The basal plane d-spacing (d_001_) at 2θ = 12.33° (calculated from Bragg’s equation) was 0.72 nm in RK. DMSO intercalation and Li cation adsorption with ultrasonic treatment reduced the H-bonding interactions between the alumina and silica layers, resulting in kaolinite exfoliation. Therefore, all the diffraction peaks of HK exhibited decreased intensity with peak broadening. Additionally, the basal plane spacing HK expanded to 1.13 nm [21].

As materials characterized by a high surface-specific area and porosity are expected to exhibit a relatively high N_2_ adsorption, the N_2_ adsorption–desorption isotherms of RK and HK were obtained and are shown in Figure 1d. Type II isotherms of the IUPAC classification system were observed for both RK and HK, suggesting that both particles were either nonporous or had a macroporous aggregate [22]. Following DMSO-exfoliation, the amount of N_2_ adsorption increased significantly with an increase in the BET surface area from 92.44 to 255.88 m^2^/g, suggesting the porosity of exfoliated kaolinite increased and more active sites for N_2_ adsorption were formed [23].

The morphologies of the RK and HK particles were examined using FESEM. The FESEM micrographs of RK and HK (Figure 1e,f) show a pseudo-hexagonal morphology in the micron range with a layered structure. The interlayer thickness of the HK particles was significantly lower, and the particles were more loosely packed than those of RK, which is consistent with the XRD results. The HK particles did not exfoliate into single-layer sheets. Therefore, the spatial morphology of HK with pores and gaps is likely to be beneficial for the pervaporation transportation of desirable components.

### 3.2. Characterization of Kaolinite–PVA Composite Membranes

**Figure 2:**
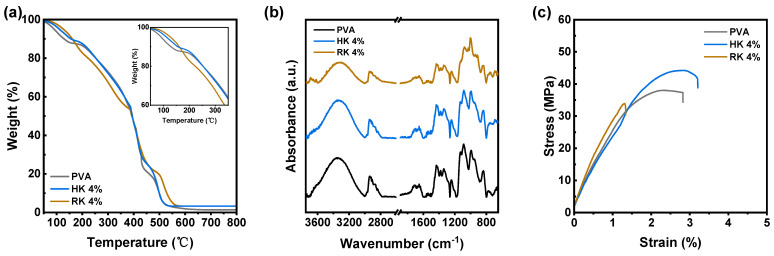
(**a**) TGA curves, (**b**) FT-IR spectra, and (**c**) stress–strain curves of PVA, PVA/HK 4 wt.%, and PVA/RK 4 wt.% MMMs.

The thermal characteristics of the PVA composite membranes provided significant insights into the distribution of particles within the polymer matrix. Therefore, a TGA study of PVA, HK/PVA, and RK/PVA membranes was performed (Figure 2a). All the membranes exhibited three noticeable weight losses. The first weight degradation that occurred from ambient temperature to 180 °C was due to the loss of absorbed water in the hydroxyl groups of the polymer matrix. However, the degradation that occurred between 190 and 480 °C was related to the elimination of hydroxyl groups and the formation of polyene macromolecules [24,25]. This was evident in the second degradation step of the RK/PVA membrane at lower temperatures compared with the PVA and HK/PVA membranes, owing to less interaction between the PVA and RK particles. Therefore, it was evident that the interaction between HK and PVA increased the thermal stability of the membrane. The third degradation step persisted after 500 °C and was related to the degradation of the main backbone of the PVA polymer, where both kaolinite composite membranes exhibited high weight loss that was ascribed to the higher stability of the inorganic residue.

The FTIR spectra of PVA, HK/PVA, and RK/PVA exhibited peaks at 3650, 2950–2850 cm^−1^, 1450 and 1650 cm^−1^, and 1078 and 837 cm^−1^ that were ascribed to the O–H stretching vibration of hydroxyl groups, CH_2_ asymmetric stretching, –CH bending vibration, and C–O stretching and C–C stretching groups, respectively (Figure 2b) [10]. Compared with the RK/PVA membrane, the HK/PVA membrane exhibited two characteristic changes owing to the interfacial interaction between HK and the PVA matrix. The first characteristic peak of OH stretching at 3650 cm^−1^ and that of the second C–O stretching at 1078 cm^−1^ exhibited an increase in the peak intensity with marginal peak shifting. While this decreased in the RK/PVA membrane, a uniform distribution of HK was observed for the PVA/HK composite. Stress–strain curves were obtained to examine the mechanical properties of the membranes (Figure 2c and Table 2). Among the three membranes, the HK/PVA membrane exhibited the highest ultimate tensile strength and elongation. Furthermore, as expected, owing to poor filler–polymer matrix interactions, the RK/PVA membrane exhibited the lowest ultimate tensile strength and elongation [26]. As corroborated by FTIR and thermogravimetric analyses, the exfoliation of HK resulted in distinct kaolinite layers, leading to an enhanced surface area and increased hydroxyl functionality. Consequently, it provided superior interaction with the PVA matrix, showcasing excellent compatibility and contributing to the augmented mechanical properties of the membranes [27]. Conversely, RK, with a reduced surface area, tends to aggregate within the matrix, resulting in inferior interfacial interactions and, consequently, lower mechanical characteristics.

**Figure 3:**
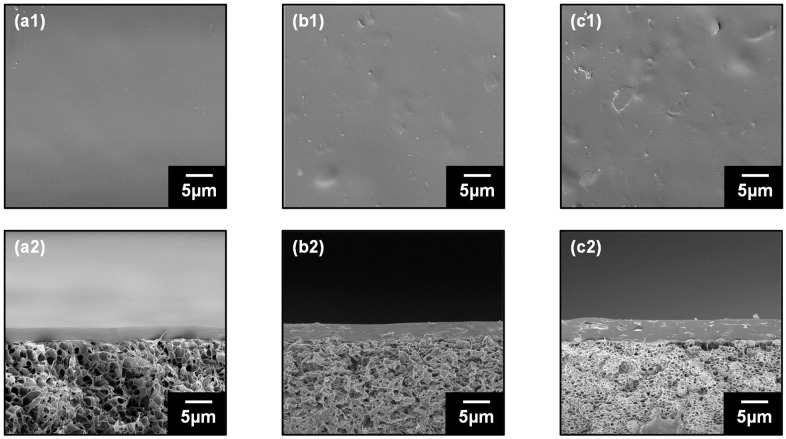
Surface (**a1**–**c1**) and cross-sectional (**a2**–**c2**) FESEM images of pristine PVA, PVA/HK 4 wt.%, and PVA/HK 10 wt.% mixed matrix membranes.

Surface and cross-sectional micrographs of the PVA and HK/PVA membranes were obtained by FESEM (Figure 3). The PVA membranes exhibited a smooth morphology with no surface defects. The HK-loaded membrane exhibited a marginally rough morphology; however, it was evident that the particles were fully accommodated within the PVA matrix, probably because of potential interactions facilitated by the elevated surface area and increased hydroxyl moieties on the HK surface. This accommodation resulted in a defect-free membrane surface. Figure 3(a2)–(c2) show that the PVA/PVDF and HK/PVA/PVDF composite membranes exhibit defect-free, thin, selective PVA or HK/PVA layers well-supported on the PVDF substrate. PVA and PVDF have strong interfacial adhesion, and no delamination of the PVA selective layer (thickness 3–4 µm) was observed.

### 3.3. Pervaporation Results

**Figure 4:**
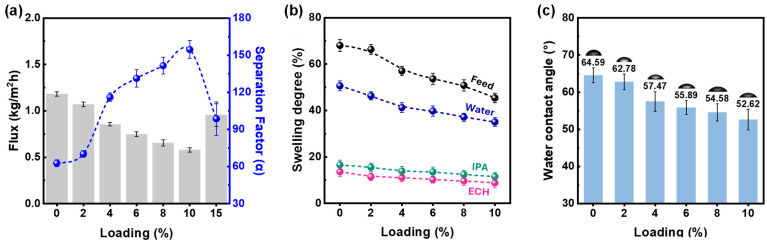
HK loading effect on (**a**) pervaporation separation performance, (**b**) swelling degree, and (**c**) contact angle on PVA/HK MMM surfaces.

Agglomeration of the RK particles occurred because of their low surface area, resulting in poor interfacial interactions with the PVA matrix, as illustrated in Figure 2. Therefore, only the PVA/HK membrane was used for the PV tests. The PVA/HK membrane was employed in the pervaporation dehydration of industrially valuable ternary feed of ECH/IPA/water (Figure 4) at 40 °C. The HK particle was loaded from 0 to 15 wt.% with respect to PVA polymer concentration. This resulted in an approximately 50% flux decrease (1.18 to 0.58 kg/m^2^h), while a three-fold (63 to 155) increase in the separation factor was observed up to an HK loading of 10 wt.%. Subsequently, the agglomeration of HK particles within the membrane led to a deterioration in the pervaporation performance observed at 15% HK loading. The swelling degree and contact angle were measured as functions of HK loading on the membrane (Figure 4b,c). Both the SD and water contact angles decreased with HK loading. The decreased water contact angle at the membrane surface suggests an increase in membrane hydrophilicity. There are two possible reasons for this as follows: first, the increased number of hydrophilic sites on the membrane surface, and second, as observed from the FESEM analysis, the enhancement in surface roughness due to the addition of inorganic particles to the membrane [9]. Figure 4b shows the effect of the SD of water, ECH, and IPA as a function of the HK loading in the membrane. The SD for water was substantially greater for all membranes than those for ECH and IPA, indicating excellent water selectivity. In addition, for the 4 wt.% HK loading, the magnitude of decrease in the SD of water was relatively less. This is reflected in Figure 4a, where the separation factor was enhanced significantly by 4% HK loading. The addition of inorganic particles that interact with the host matrix generally increases the rigidity of the membrane, thereby reducing its free volume for pervaporation transportation [27,28]. This is because the flux linearly decreases with HK particle loading. A similar observation was made in the study by Wang et al., who used UiO-66 in the PVA matrix; the flux decreased with an increase in the separation factor for butanol pervaporation dehydration, which was attributed to the less hydrophilic nature of UiO-66 [28]. Furthermore, Raesi et al. explored a titanate nanotube as a filler in a PVA matrix for the pervaporation of isopropanol and water mixtures, and a reduction in flux with increasing separation factor was observed, confirming that the observed phenomena were related to the enhancement in membrane rigidity due to the PVA-Ti nanotube interaction [29]. However, upon comparing the magnitudes of the flux reduction and enhancement in the separation factor, it is evident that the hydrophilic modification of kaolinite effectively facilitates water permeation, demonstrating an anti-trade-off phenomenon between the flux and separation factor. As HK has a basal plane d-spacing of 0.72 nm and also has Al-OH, it enables the transit of the water molecule by providing a smooth path through the membrane. However, ECH and IPA are larger and less polar than water; therefore, they resist this movement of water.

HK with a loading of 4 wt.% exhibited the highest PV performance. Hence, it was chosen for further evaluation of PV performance under varying operating parameters.

**Figure 5:**
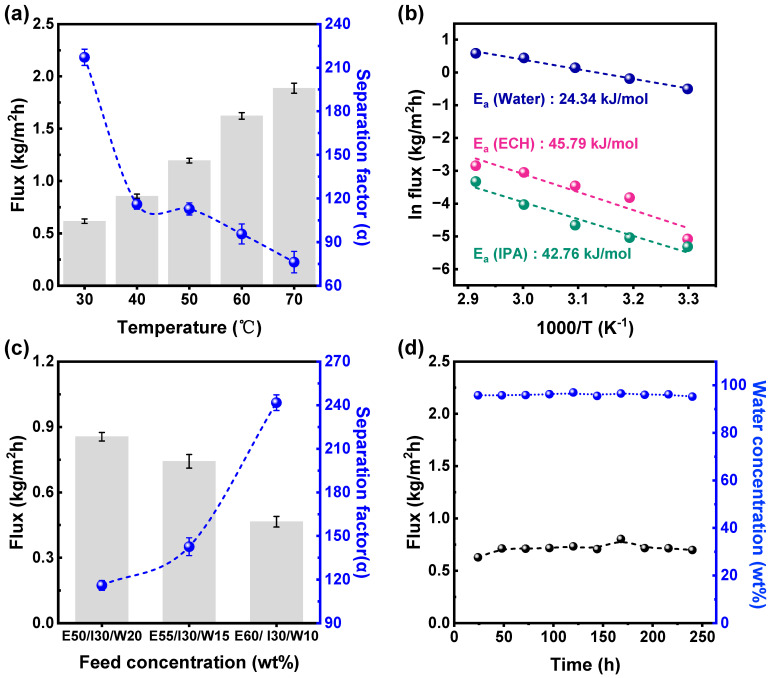
(**a**) Effect of the feed temperature on the pervaporation performance, (**b**) plot of ln flux vs. 1000/temperature, (**c**) feed concentration, and (**d**) long-term stability test using the PVA/HK (4 wt.%) membrane with ECH/IPA/water (50/30/20 *w*/*w* %) at 40 °C.

The feed temperature was increased from 30 to 70 °C, and the PV test was performed with ECH/IPA/water at a 50/30/20 (*w*/*w*, %) feed mixture composition. The flux increased linearly with feed temperature, but a three-fold decrease in the separation factor was observed (Figure 5a). Additionally, the decrease in the separation factor was more pronounced between 30 and 40 °C; thereafter, it decreased linearly. The increase in flux can be attributed to the enhanced driving force (vapor pressure) of the components. Furthermore, the thermal agitation of the polymer chain enhances the free volume across the PVA membrane, thereby increasing the rate at which permeating components are transported [8,9,10]. The sharp decrease in the separation factor between 30 and 40 °C suggested that the membranes produced in this study were sensitive to the feed temperature. This is because, as observed in the XRD study, the basal plane spacing (0.72 nm) in the HK sheet was sufficient to enable the transportation of all three components without manifestation of the sieving effect; however, spatially, the hydrophilicity of the membrane system resulted in satisfactory separation performance. However, increasing the temperature increased the vapor pressures of ECH, IPA, and water at the membrane boundary layer. This resulted in the adsorption of ECH- and IPA-rich water molecules onto the membrane surfaces and their subsequent diffusion through the expanded PVA chain. The temperature-dependent flux of the PVA-based membranes was characterized using the Arrhenius equation [30,31].

(4) $$Flux\;\left(Ji\right)=Ap\times e^{-EP/RT},$$

where *Ji* (kg m^−2^ h^−1^) is the flux of component i (ECH, IPA, water), and T (K), R (J mol^−1^ K^−1^), *A*_p_ (kg m^−2^ h^−1^) and *E*_p_ (kJ mol^−1^) are the absolute temperatures, molar gas constant, pre-exponential factor, and apparent energy of activation for permeation, respectively.

The apparent activation energies for the permeation of ECH, IPA, and water were calculated from the slopes of the logarithmic plots of their individual fluxes as a function of 1000 divided by the temperature. The *E_P_* values for ECH (45.79 kJ/mol) and IPA (42.76 kJ/mol) were higher than that of water (24.34 kJ/mol), as depicted in Figure 5b. This phenomenon suggests that the permeation of IPA and ECH is more sensitive to the feed temperature. ECH and IPA have a high transportation barrier because their molecular sizes are higher than that of water. However, with an increase in the feed temperature, the transportation barrier was significantly reduced, and the rates of permeation of ECH and IPA were higher than that of water; therefore, the separation factor decreased.

Figure 5c shows the effect of the feed composition on the PV performance of the kaolinite–PVA composite membranes. The water and ECH contents varied from 20 to 10 wt.% and 50 to 60 wt.% in the feed, respectively, at 40 °C. The flux reduced from 0.86 to 0.47 kg/m^2^h (50% reduction), while the separation factor was enhanced from 116 to 242 (2-fold increase) with a decrease in the water content in the feed. The increased flux and decreased separation factor with water in the feed were attributed to the plasticization effect imposed by the water molecules on the PVA polymer chain, which exhibited high swelling and higher spacing during pervaporation molecular transport [32]. However, compared with other mixed-matrix membranes, the reduction in the separation factor was less profound [8,10]. As observed in the aforementioned test, the PVA-HK interaction marginally increased the rigidity of the membrane, which decreased membrane swelling. However, HK had sufficient basal-plane d-spacing, which might have acted as a hydrophilic channel for significant water permeation. Therefore, the separation tended to decrease less significantly with an increase in feed water content.

The long-term durability of the membranes is an important parameter for studying their industrial applicability. Figure 5d shows the results of the 250 h long-term operating test for the PVA/HK 4% membrane with feed containing ECH/IPA/water (50/30/20, *w*/*w*%) at 40 °C. The feed composition was maintained throughout the operating period, and the intermittent flux and water content of the permeate solution were evaluated. During the entire operating time, neither of the membrane performance evaluation parameters deviated considerably. This indicates that the incorporation of HK into the membrane significantly limited the membrane swelling of the PVA polymer chain. Additionally, the HK–PVA composite membrane exhibited excellent resistance to electrophilic ECH-reactive molecules.

Our research group is the first to perform pervaporation separation of azeotropic ECH-IPA-water mixtures. Table 3 presents a comparative analysis of pervaporation performance across various membranes for the ECH–IPA–water systems. Compared with our previously developed polymer–alumina composite membranes, the PVA/HK composite membrane developed in this study delivered a moderate flux and achieved a relatively lower separation factor. Therefore, further efforts should be made to modify the HK to enhance the hydrophilicity of the filler, thereby enhancing the separation factor in future work.

## 4. Conclusions

In this study, the use of raw kaolinite led to particle agglomeration in the PVA matrix. Consequently, DMSO exfoliation of kaolinite was conducted to enhance the hydrophilicity of the kaolinite particles. The exfoliation of kaolinite decreases the Si/Al ratio (1.18 to 1.16) and increases the basal plane spacing to 1.13 nm and the particle surface area to 255.88 m^2^/g, as confirmed by XPS, XRD, and BET analyses, respectively. The RK particles agglomerated in the PVA matrix, whereas the HK particles showed excellent dispersibility in the PVA matrix. The HK/PVA selective layer coated on the PVDF support membrane was examined for dehydration of the ECH/IPA/water mixture under various operating conditions. A membrane with four wt.% HK with respect to the PVA concentration demonstrated the highest pervaporation dehydration ability (flux = 0.86 kg/m^2^h and separation factor 116) in the processing of ECH/IPA/water feed mixture dehydration. Furthermore, in the operating condition tests, the total pervaporation flux of the HK/PVA membrane increased with increasing feed temperature and water content, whereas the separation factor decreased. The water selectivity of the HK/PVA composite membrane arose from hydrophilicity enhancement and the smooth water channels produced in the PVA matrix due to the inherent hydrophilic basal spacing. The incorporation of HK into the PVA membrane demonstrates its great potential for ECH dehydration. Hence, this one-pot hydrophilic modification technique for HK presents a practical approach for improving particle dispersion in polymer matrices.

## Figures and Tables

**Table 1:** Atomic percentages of elements based on the survey spectra of kaolinite particles following XPS.

Particle Type	Elements	Wt.%	Atomic %
RK	O	54.18	67.09
	Al	20.71	15.21
	Si	25.10	17.70
HK	O	55.24	68.03
	Al	20.24	14.78
	Si	24.52	17.20

**Table 2:** Mechanical properties of the PVA, PVA/HK, and PVA/RK composite membranes.

Samples	Tensile Strength (MPa)	Elongation (%)
PVA	37.34	2.82
HK 4 wt.%	41.59	3.20
RK 4 wt.%	32.26	1.34

**Table 3:** Comparison of the pervaporation performance of different membrane systems of the ECH–IPA–water feed system.

Matrix	Nature of the Membrane Preparation Method	Feed (%) Composition, (*w*/*w*)	Temperature, (^o^C)	Flux (J) (kg/m^2^h)	Separation Factor (∝)	Thickness (µ)	Reference
PVA	Blending with GO	ECH/IPA/water 50/30/20	40	0.13	4670	15	[33]
PVA	CMC40-AHF	ECH/IPA/water 50/30/20	40	1.214	638	7.7	[34]
PVA	Kaolinite–PVDF support	ECH/IPA/water 50/30/20	40	0.86	166	3–4	[This study]

## Data Availability

The data are contained within this article.
